# Perioperative outcomes after thoracic aortic surgery in Johannesburg: a retrospective observational cohort study, 1995–2023

**DOI:** 10.1016/j.bjao.2026.100891

**Published:** 2026-09-22

**Authors:** Maserufe Marcia Mmabatswa, Amanda Nkuna, Kathy Vanderdonck, Moses Mogakolodi Kebalepile, Palesa Motshabi-Chakane

**Affiliations:** Department of Anaesthesiology, Faculty of Health Sciences, University of the Witwatersrand, Johannesburg, South Africa

**Keywords:** aortic aneurysm, aortic dissection, HIV, perioperative outcomes, South Africa, thoracic aortic surgery, trauma

## Abstract

**Background:**

Thoracic aortic diseases require complex surgery, and outcomes data from African populations are scarce. We described the patient characteristic profile, documented risk factors, and perioperative outcomes of patients undergoing thoracic aortic surgery at a quaternary hospital in South Africa.

**Methods:**

In a retrospective cohort study reported per the Strengthening the Reporting of Observational Studies in Epidemiology guidelines, we included patients aged 18 yr or older who underwent open thoracic aortic surgery at Charlotte Maxeke Johannesburg Academic Hospital between 1995 and 2023. The primary outcome was in-hospital mortality. We assessed correlates using modified Poisson regression with robust variance (risk ratios [RRs]).

**Results:**

Of 366 patients (median age 48 yr [inter-quartile range 37–59]; 219 [59.8%] male), diagnoses were dissection in 157 (42.9%), aneurysm in 157 (42.9%), traumatic rupture in 26 (7.1%), and other in 26 (7.1%). In-hospital mortality was 21.9% (80 of 366; 95% confidence interval [CI] 17.7–26.4) and did not differ significantly across diagnosis groups (*P*=0.693), being identical after dissection and aneurysm (22.9% in each). Prolonged operative duration (>600 min) was the only variable independently associated with mortality (adjusted RR 7.2; 95% CI 1.5–34.9), an imprecise, sparse-data estimate. Mortality was numerically higher in female than male patients (26.2% *vs* 19.2%) but this difference was not significant after adjustment (adjusted RR 1.30; 95% CI 0.89–1.89); age and diagnosis were not independently associated.

**Conclusions:**

In-hospital mortality after thoracic aortic surgery was 21.9%. Prolonged operative duration was associated with in-hospital mortality, although the estimate was imprecise, and operative duration is likely to be a marker of procedural complexity and adverse intraoperative course rather than an independently modifiable risk factor. The findings support intensive postoperative care.

**Editor’s key points**

• Most published data on thoracic aortic surgery come from high-income countries. This study offers long-term real-world evidence from an African quaternary hospital with different patients, disease patterns, resources, and referral pathways.
• In 366 patients, in-hospital mortality was 21.9%. These findings are important for guiding quality improvement projects, patient counselling, service planning, and outcome benchmarking.
• The cohort included many patients with aortic dissection and aneurysm, plus younger patients with traumatic aortic rupture, reflecting regional disease patterns less often seen in Western series.
• Very long operations were associated with mortality, likely reflecting greater operative complexity rather than proving that duration itself causes death.
• The limitations of this retrospective audit highlight the need to improve future surgical registries and hospital record systems.

The aorta is the principal conduit for delivering oxygenated blood, and diseases affecting it, such as aneurysms and dissections, are catastrophic and often necessitate high-risk surgical intervention.^1^^,^^2^ Without surgery, mortality is exceedingly high.^3^ Global studies have documented improvements in outcomes for thoracic aortic surgery, attributed to advances in surgical technique, perioperative care, and centralisation of services.^4^^,^^5^

The epidemiological landscape and patient risk profiles in sub-Saharan Africa are distinct.^6^ South Africa contends with a high burden of multiple diseases, including a high prevalence of human immunodeficiency virus (HIV), which has been linked to aortic aneurysms through HIV-associated vasculitis,^7^, ^8^, ^9^ and a substantial trauma burden.^10^ Despite this, comprehensive data on the presentation and outcomes of thoracic aortic surgery from the African continent remain limited.^6^^,^^11^

This study aimed to describe the patient characteristic profile, documented risk factors, and perioperative outcomes of patients undergoing thoracic aortic surgery at Charlotte Maxeke Johannesburg Academic Hospital (CMJAH) between January 1995 and December 2023, with in-hospital mortality as the primary outcome. Patient characteristics and perioperative correlates of mortality were assessed by multivariable regression.

## Methods

### Study design, site, and population

We conducted a retrospective observational cohort study at CMJAH, a quaternary referral centre in Johannesburg, South Africa, managing complex thoracic aortic surgical cases. The study population comprised all adult patients aged 18 yr or older who underwent open thoracic aortic surgery between January 1995 and December 2023. We identified patients from departmental databases and hospital records. We excluded patients who underwent non-thoracic aortic procedures, were younger than 18 yr, or had missing diagnosis data required for classification. The study included all eligible adult patients undergoing open thoracic aortic surgery at CMJAH during the predefined study period; therefore, we did not perform an *a priori* sample-size calculation.

### Data sources

We retrieved data for 1995–2016 from an existing electronic database maintained by the Department of Anaesthesiology at CMJAH. For 2017–23, we collected data through retrospective review of patient medical records. Data extracted included patient characteristics, clinical presentation, perioperative variables, and postoperative outcomes. We assessed data quality by re-abstracting a random sample of 22 patient records and comparing each captured value with the source record. We selected records at random by file number and retrieved the corresponding source files from the hospital records department. We identified discrepancies in eight of the 22 records (36.4%). In every instance, the discrepancy involved documented pre-existing risk factors: risk factors recorded in the source file, principally hypertension and HIV, were absent from the captured dataset, and this occurred more often among records drawn from the earlier electronic database than among those abstracted directly from the medical records. In two of these eight records, the admission and discharge dates and the duration of intensive care stay were also present in the source file and absent from the electronic database. The captured dataset was not amended after the audit, so the analyses presented here use the data as originally captured; the limitations address the implications. Patient characteristic and clinical variables included age, sex, primary diagnosis (dissection, aneurysm, traumatic aortic rupture, or other pathology), and documented pre-existing risk factors (hypertension, HIV, trauma, connective tissue disorder, and infection). Traumatic aortic dissections were classified under the traumatic aortic rupture category. All diagnoses were made clinically by surgeons and subsequently confirmed radiologically with computed tomography (CT) imaging.

### Outcome measures and definitions

The primary outcome was in-hospital mortality. Postoperative complications assessed were haemorrhage, respiratory failure, renal failure, neurological deficit, sepsis, cardiac arrest, clinically significant arrhythmia, heart failure requiring mechanical circulatory support, and extracorporeal membrane oxygenation use. We applied the following definitions consistently throughout the manuscript and tables. Haemorrhage was recorded as a composite of two separately coded bleeding events: reoperation for bleeding and haemorrhage defined as blood loss requiring a massive transfusion protocol. A patient coded for either event, or for both, contributes a single event to this category, so the composite count is smaller than the sum of the two component counts, which would double-count patients coded for both. Respiratory failure was defined as postoperative respiratory failure requiring prolonged mechanical ventilation (>24 h), re-intubation, or prolonged ventilatory support, per the Society of Thoracic Surgeons.^12^ Renal failure was defined as documented postoperative renal dysfunction requiring renal replacement therapy. Neurological deficit was defined as stroke, spinal cord ischaemia, or neurological dysfunction persisting at discharge. We defined significant arrhythmia as arrhythmia requiring a permanent pacemaker. Follow-up was limited to the index hospital admission; we did not include post-discharge data. We ascertained in-hospital mortality from the clinical mortality outcome field of the source record. This field was cross-checked against a parallel numeric mortality-code field and any recorded date of death. Six recorded dates of death (showing intervals >90 days postoperatively, including up to 20 yr) were identified as data-capture errors resulting from date misformatting or transcription of the year. Source-file verification confirmed that all six were true in-hospital deaths during the index admission, leaving total in-hospital mortality unchanged (80 of 366). These six records were excluded from exploratory death-timing assessments.

### Statistical analysis

We summarised categorical variables using frequencies and percentages, and continuous variables using medians and inter-quartile ranges (IQRs). We reported proportions with 95% confidence intervals (CIs) using the Wilson method. Group comparisons for categorical variables were performed using Fisher’s exact test (evaluated by complete enumeration of tables with observed margins), because several expected cell counts across the four diagnosis groups were less than five. We reported missing data per variable rather than assuming any particular mechanism. Diagnosis, age, and mortality were complete; ICU stay was available for 336 of 366 (91.8%) and operative duration for 343 of 366 (93.7%). Risk-factor documentation was missing for 194 of 366 (53.0%) and hospital length of stay for 244 of 366 (66.7%); these two variables were reported descriptively with explicit denominators and were not used as adjustment covariates. We did not assert that missingness was completely at random. The multivariable model used complete cases for the included covariates (*n*=341, 76 deaths); we state this as a limitation.

We assessed factors associated with in-hospital mortality using modified Poisson regression with robust (sandwich) variance, which directly estimates relative risks and is more stable than log-binomial regression when the latter fails to converge.^13^^,^^14^ We specified candidate covariates *a priori* on clinical grounds and entered them into the model together. Continuous variables were categorised based on clinical risk strata (age, 18–29, 30–39, 40–49 [reference], 50–59, and ≥60 yr; operative duration, short [≤300 min, reference], moderate [301–600 min], and prolonged [>600 min]). No univariable screening was performed, and no data-driven variable selection of any type was used; the model was fixed before the outcome data were examined and was not subsequently modified. The model comprised 10 coefficients on 76 deaths, corresponding to approximately 7.6 events per variable, which is below the conventional threshold of 10; this is acknowledged as a limitation, and the estimates should be interpreted with corresponding caution. Because cardiopulmonary bypass, cross-clamp, and total operating-room time were strongly correlated, we retained only total operating-room time. Statistical significance was defined as two-sided *P*<0.05. We performed analyses in Python 3 (*statsmodels, lifelines*) and cross-checked them against the original Stata (StataCorp, College Station, TX, USA) outputs.

No time-to-event analysis of in-hospital mortality was undertaken. A previous version of this analysis described the temporal pattern of in-hospital death using cumulative incidence functions on a time scale of days on ICU, treating live discharge from ICU as the competing event. That framework requires that a patient discharged alive from ICU can no longer experience the endpoint. On re-audit of the source records, this condition does not hold in this cohort: among deaths for which the date of death, the date of operation, and the ICU stay were all recorded, a substantial minority occurred after the patient had left ICU. Re-framing the analysis on time to in-hospital death with live hospital discharge as the competing event, as would be preferable, was not possible because discharge dates are recorded for only a minority of survivors. Therefore, we withdrew the cumulative incidence analysis and its figures and reported mortality as a proportion.

### Ethical considerations

The University of the Witwatersrand Human Research Ethics Committee (Medical) granted ethical approval (M240844), with a waiver of individual informed consent for this retrospective record review. We anonymised all data and stored them securely. The study was registered with the National Health Research Database (GP_202404-014). The study was conducted and is reported in accordance with the Strengthening the Reporting of Observational Studies in Epidemiology guidance for cohort studies.^15^

## Results

### Participant characteristics

We retrieved 456 patient records. After screening, 90 records were excluded: 28 (6.1%) non-thoracic aortic procedures, 5 (1.1%) with missing age, 3 (0.7%) aged <18 yr, and 54 (11.8%) with missing diagnosis. The final analytical cohort comprised 366 patients (Fig. 1).

**Fig 1:**
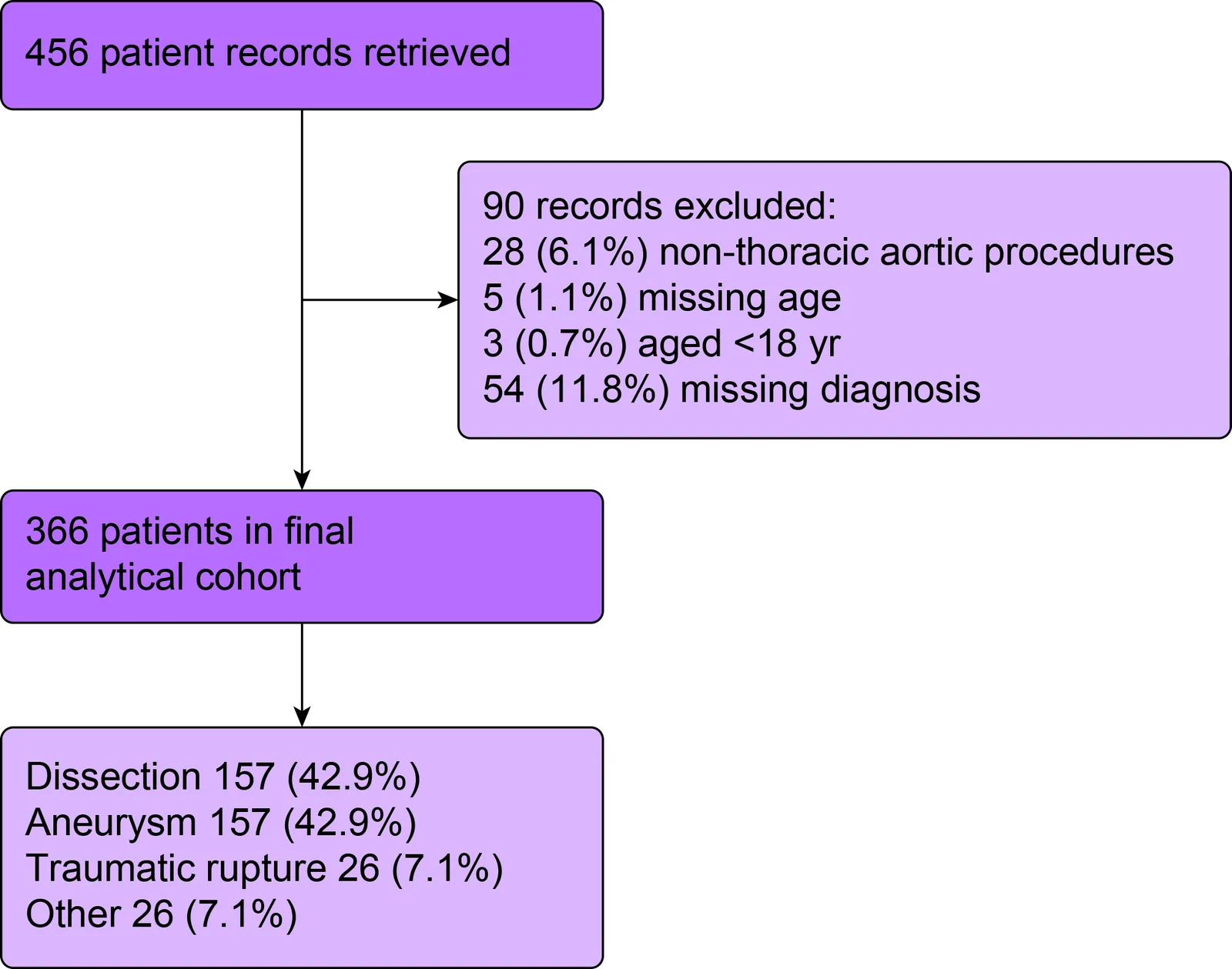
Study flow diagram showing record retrieval, exclusions, and the final analytical cohort of 366 patients.

Aortic dissection and aneurysm were the two dominant indications and occurred in equal numbers (157 of 366, 42.9% each), followed by traumatic aortic rupture (26 of 366, 7.1%) and other thoracic aortic pathologies (26 of 366, 7.1%) (Table 1). The overall median (IQR) age was 48.0 yr (37.0–59.0). Patients with traumatic aortic rupture and other diagnoses were substantially younger (median 30 and 31 yr) than patients with dissection (54 yr) or aneurysm (47 yr). Overall, 219 of 366 patients (59.8%) were male, and 145 of 366 (39.6%) were female (sex was undocumented for 2 [0.5%]). Male predominance was most marked among patients with traumatic aortic rupture (25 of 26, 96.2%). The median (IQR) ICU stay was 4.0 days (2.0–7.0), longest after dissection surgery (5.0 days [3.0–10.0]). Hospital length-of-stay data were available for 122 of 366 patients (33.3%). Risk-factor status was documented for 172 of 366 patients (47.0%) and undocumented for 194 of 366 (53.0%); therefore, risk-factor frequencies are reported over the number of patients with a documented record rather than the whole cohort. Among those 172 patients, hypertension was the most documented risk factor (71 of 172, 41.3%), followed by HIV (50 of 172, 29.1%) and trauma (45 of 172, 26.2%). These counts include risk factors recorded in any of the record’s risk-factor fields; a previous version counted only the first field, which undercounted every category.

**Table 1:** Baseline patient, perioperative, and clinical characteristics of patients undergoing thoracic aortic surgery at Charlotte Maxeke Johannesburg Academic Hospital, 1995–2023. Percentages for age, sex, ICU stay, CPB, and operating-room time are within each diagnosis column. Risk factors are reported as the number positive over the number of patients in that column with any documented risk-factor record, because risk-factor documentation is absent for a substantial minority and those patients can contribute neither a positive nor a negative. A patient may carry more than one risk factor, so the risk-factor rows are not mutually exclusive and do not sum to the documented total. Hospital length of stay is not tabulated: it was recorded for a minority of patients and the recorded values are unreliable. Data completeness by column for continuous perioperative variables: ICU stay was available for 145/157 dissection, 142/157 aneurysm, 25/26 traumatic rupture, and 24/26 other. CPB, cardiopulmonary bypass; HIV, human immunodeficiency virus; IQR, inter-quartile range; OR, operating room.

Characteristic	Dissection (*n*=157)	Aneurysm (*n*=157)	Traumatic rupture (*n*=26)	Other (*n*=26)	Total (*N*=366)
Age, median (IQR)	54 (44–61)	47 (37–59)	30 (27–39)	31 (24–53)	48 (37–59)
Male sex, *n* (%)	78 (49.7)	94 (59.9)	25 (96.2)	22 (84.6)	219 (59.8)
Female sex, *n* (%)	78 (49.7)	62 (39.5)	1 (3.8)	4 (15.4)	145 (39.6)
Sex undocumented, *n* (%)	1 (0.6)	1 (0.6)	0 (0.0)	0 (0.0)	2 (0.5)
ICU stay (days), median (IQR)	5 (3–10)	4 (2–5)	3 (2–5)	2 (1–3)	4 (2–7)
CPB time (min), median (IQR)	204 (142–287)	203 (140–277)	100 (37–131)	146 (122–267)	200 (135–276)
Total OR time (min), median (IQR)	480 (401–615)	465 (390–560)	312 (259–352)	345 (311–438)	465 (374–590)
Hypertension, *n*/*N* documented (%)	41/54 (75.9)	29/80 (36.2)	0/24 (0.0)	1/14 (7.1)	71/172 (41.3)
HIV, *n*/*N* documented (%)	11/54 (20.4)	38/80 (47.5)	1/24 (4.2)	0/14 (0.0)	50/172 (29.1)
Trauma, *n*/*N* documented (%)	1/54 (1.9)	11/80 (13.8)	24/24 (100.0)	9/14 (64.3)	45/172 (26.2)
Connective tissue disorder, *n*/*N* documented (%)	4/54 (7.4)	9/80 (11.2)	0/24 (0.0)	1/14 (7.1)	14/172 (8.1)
Infection, *n*/*N* documented (%)	2/54 (3.7)	7/80 (8.8)	0/24 (0.0)	1/14 (7.1)	10/172 (5.8)
Risk-factor status undocumented, *n* (%)	103 (65.6)	77 (49.0)	2 (7.7)	12 (46.2)	194 (53.0)

### Distribution of diagnoses over time

The distribution of diagnoses varied across the study period (Fig. 2). Aortic dissection and aneurysm accounted for most procedures throughout. Dissection was the more common indication in the earlier and middle periods (64 of 150, 43% of procedures performed in 1995–2005, and 41 of 86, 48% in 2006–15), whereas aneurysm repair predominated in the most recent period (71 of 128, 55% in 2016–23). Traumatic aortic rupture accounted for a smaller proportion that declined across the period (13 of 150, 9% in 1995–2005, to 2 of 128, 2% in 2016–23), and the other category remained uncommon.

**Fig 2:**
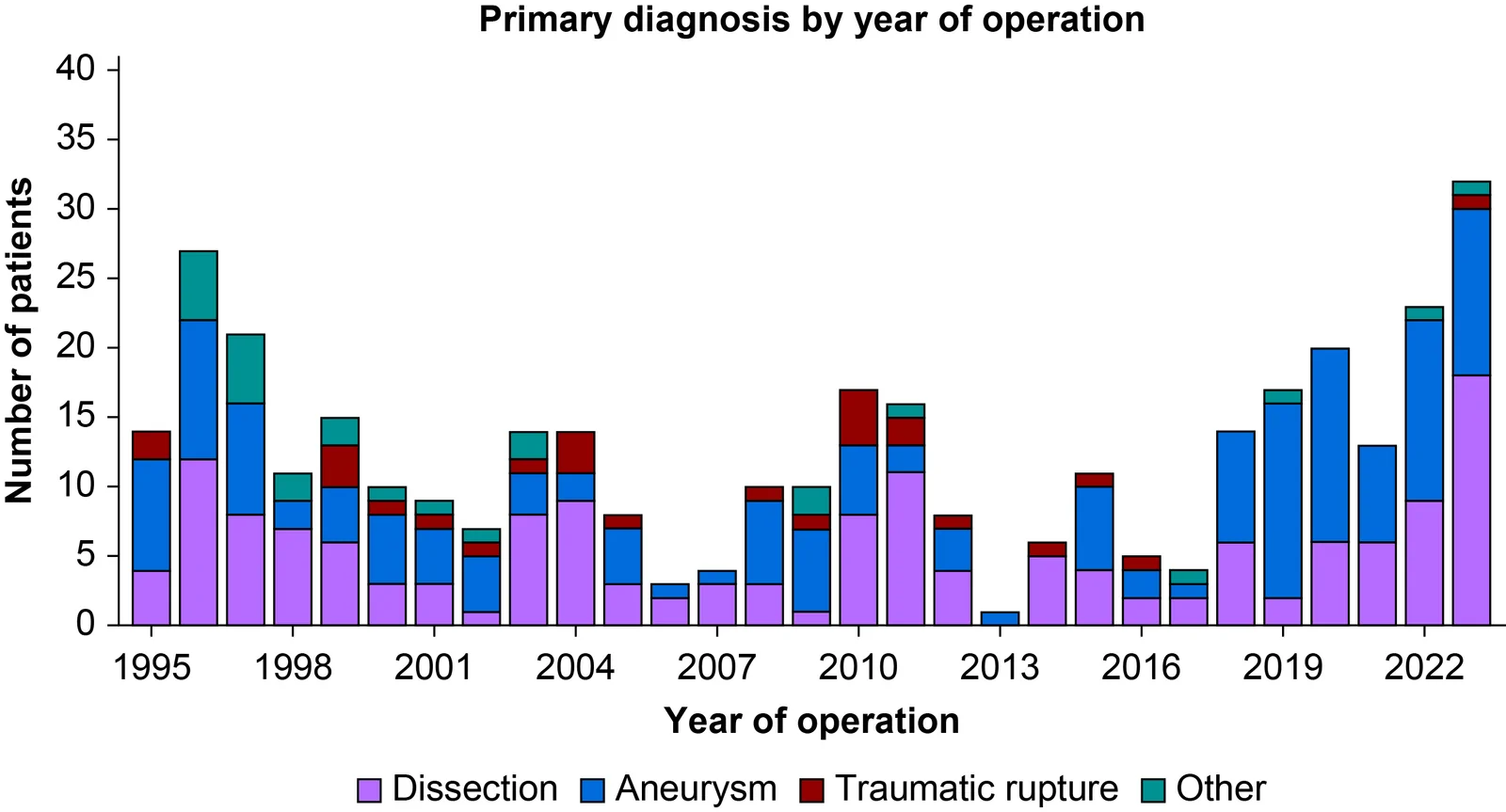
Distribution of primary diagnosis by year of operation, 1995–2023 (stacked annual counts).

### Postoperative complications and mortality

In-hospital mortality was 21.9% (80 of 366; 95% CI 17.7–26.4). Mortality did not differ significantly across diagnosis groups (*P*=0.693) and was identical after dissection and aneurysm repair (36 of 157, 22.9% in each); it was lower, in small numbers, after traumatic rupture and in the other category (4 of 26, 15.4% in each) (Table 2). Haemorrhage was the most frequent complication (76 of 366, 20.8%) and did not differ significantly across diagnosis groups (*P*=0.236); unlike the other complications, it was numerically most common after aneurysm repair (38 of 157, 24.2%) rather than after dissection (32 of 157, 20.4%). Respiratory failure (31 of 366, 8.5%) and renal failure (29 of 366, 7.9%) were the next most frequent and were numerically more common after dissection repair, but neither difference reached significance across diagnosis groups (*P*=0.100 and *P*=0.102, respectively); neurological deficit was the only complication that differed significantly (*P*=0.049), being most frequent after dissection (15 of 157, 9.6%). Because several cells in Table 2 have expected counts of less than five, all group comparisons are done using Fisher’s exact test; the neurological-deficit result lies close to the conventional threshold and rests on sparse cells and is therefore not relied upon in the interpretation below.

**Table 2:** Postoperative complications and in-hospital mortality by diagnosis group. Values are *n* (%) within each diagnosis column. Haemorrhage is a combined category comprising two separately coded bleeding events, reoperation for bleeding and haemorrhage, counted once per patient: patients coded for both contribute a single event, so the combined figure is lower than the sum of the two component counts. Respiratory failure: prolonged ventilatory support (>24 h) or re-intubation. Renal failure: documented postoperative renal dysfunction requiring renal replacement therapy. Neurological deficit: stroke, spinal cord ischaemia, or neurological dysfunction persisting at discharge. *P*-values compare each complication across the four diagnosis groups using Fisher’s exact test, computed by complete enumeration of all tables with the observed margins; an exact test was used in place of the χ^2^ approximation because several cells have expected counts of less than five.

Complication	Total (*N*=366)	Dissection (*n*=157)	Aneurysm (*n*=157)	Traumatic rupture (*n*=26)	Other (*n*=26)	*P*-value
Haemorrhage	76 (20.8)	32 (20.4)	38 (24.2)	4 (15.4)	2 (7.7)	0.2363
Respiratory failure	31 (8.5)	19 (12.1)	11 (7.0)	1 (3.8)	0 (0.0)	0.0998
Renal failure	29 (7.9)	18 (11.5)	10 (6.4)	0 (0.0)	1 (3.8)	0.1016
Neurological deficit	21 (5.7)	15 (9.6)	5 (3.2)	0 (0.0)	1 (3.8)	0.0488
Sepsis	21 (5.7)	10 (6.4)	10 (6.4)	0 (0.0)	1 (3.8)	0.6126
In-hospital mortality	80 (21.9)	36 (22.9)	36 (22.9)	4 (15.4)	4 (15.4)	0.6932

### Factors associated with in-hospital mortality

In the modified Poisson regression, prolonged operative duration (>600 min) was the only variable independently associated with in-hospital mortality (adjusted risk ratio [RR] 7.2; 95% CI 1.5–34.9) (Table 3). The CI is wide, reflecting sparse deaths across the operative-duration strata and near-separation, so the estimate should be interpreted with caution; it is best read as a marker of operative complexity and intraoperative course rather than a directly modifiable target. Mortality was numerically higher among female than male patients (26.2% *vs* 19.2%), but the difference was not statistically significant after adjustment (adjusted RR 1.30; 95% CI 0.89–1.89). Neither age category nor primary diagnosis was independently associated with mortality.

**Table 3:** Factors associated with in-hospital mortality (modified Poisson regression with robust variance; complete-case *n*=341, 76 deaths). Estimated by modified Poisson regression with robust (sandwich) variance, complete-case for the included covariates. The CI for prolonged operative duration is wide, reflecting sparse deaths across operative-duration strata; interpret the estimate with caution. RR, risk ratio; CI, confidence interval.

Variable	Category	Crude RR (95% CI)	Adjusted RR (95% CI)	*P*-value (adjusted)
Age	18–29 yr	1.45 (0.69–3.02)	1.88 (0.86–4.07)	0.111
	30–39 yr	1.39 (0.70–2.76)	1.87 (0.94–3.71)	0.072
	40–49 yr	Reference	Reference	-
	50–59 yr	1.22 (0.63–2.36)	1.39 (0.74–2.62)	0.304
	≥60 yr	1.76 (0.96–3.20)	1.75 (0.98–3.12)	0.058
Sex	Male	Reference	Reference	-
	Female	1.38 (0.93–2.05)	1.30 (0.89–1.89)	0.176
Diagnosis	Dissection	Reference	Reference	-
	Aneurysm	1.00 (0.66–1.50)	1.06 (0.71–1.59)	0.759
	Traumatic rupture	0.58 (0.20–1.73)	1.15 (0.29–4.52)	0.846
	Other	0.53 (0.14–2.01)	0.62 (0.17–2.22)	0.462
Operative duration	Short (≤300 min)	Reference	Reference	-
	Moderate (301–600 min)	2.74 (0.70–10.77)	2.93 (0.62–13.84)	0.174
	Prolonged (>600 min)	6.56 (1.67–25.69)	7.20 (1.48–34.92)	0.014

## Discussion

The main findings of this single-centre review of surgery performed between 1995 and 2023 are that one in five patients died in hospital after thoracic aortic surgery, that prolonged operative duration was the only variable independently associated with mortality, and that neither sex, age, nor diagnostic category was independently associated with mortality once modelled with a stable estimator. The cohort was characterised by an equal burden of dissection and aneurysm and a distinct sub-population of young patients with traumatic rupture.

The overall in-hospital mortality of 21.9% is higher than contemporary reports from high-income countries, which often cite rates of less than 15% for elective repair.^4^^,^^16^ This likely reflects the complex case mix at a quaternary referral centre, including a high proportion of emergency dissections and ruptures, delayed presentations, resource constraints, and comorbid disease. Hypertension was common among patients with dissection and aneurysm, consistent with the established role of chronic vascular disease in thoracic aortic pathology.^16^ HIV was documented in 38 of the 80 patients with aneurysm and a documented risk-factor record (47.5%); because risk-factor documentation was missing for more than half the cohort, HIV could not be modelled, and this observation is descriptive only and should not be read as an adjusted association.^17^^,^^18^

Prolonged operative duration was the variable most strongly associated with mortality. Longer operating room, cardiopulmonary bypass, and cross-clamp times are surrogates for surgical complexity and prolonged exposure to the extracorporeal circuit, with attendant systemic inflammation, coagulopathy, and end-organ injury.^19^^,^^20^ The association is imprecise and likely reflects operative complexity and the intraoperative course, including reverse causation in which an intraoperative catastrophe prolongs surgery, rather than a directly modifiable target; therefore, it should not be read as evidence that shortening operative time alone would reduce mortality. The organ complications (respiratory, renal, and neurological) remained numerically most frequent after dissection, and neurological deficit significantly so; this is consistent with malperfusion physiology, in which propagation of the false lumen compromises coronary, cerebral, mesenteric, renal, or spinal perfusion.^21^^,^^22^ Haemorrhage did not follow that pattern and was numerically most common after aneurysm repair, consistent with the extent of aortic reconstruction and the associated coagulopathy rather than with malperfusion. However, mortality itself did not differ across diagnosis groups.

Our analysis did not confirm independent associations with sex or age. Mortality was numerically higher among female patients, but this did not reach significance after adjustment, and we caution against over-interpreting a difference that the data do not support. This restraint aligns with the study’s descriptive aim.

### Limitations

This study has limitations inherent to its retrospective design. Risk-factor documentation, including HIV status, was missing for more than half the cohort, and hospital length-of-stay data were largely incomplete; these variables could not be included in the multivariable model, so no adjusted inference about HIV or other documented risk factors is possible. The re-abstraction audit indicates that this under-documentation is not confined to records in which no risk factor was recorded: risk factors present in the source file were absent from the captured dataset in eight of the 22 records re-abstracted, more often among records drawn from the earlier electronic database. Therefore, the documented risk-factor frequencies reported here, including HIV in 50 of 172 patients, are likely to underestimate the true prevalence in this cohort, and the discrepancies identified by the audit were not reconciled in the analysis dataset. The multivariable model used complete cases for the included covariates and comprised approximately 7.5 events per variable, below the conventional threshold of 10, so its estimates are modestly imprecise. No time-to-event analysis is presented. Dates of death and of hospital discharge were too incompletely recorded to support one: a cumulative incidence analysis using ICU discharge as the competing event was withdrawn because in-hospital deaths were found to occur after ICU discharge in this cohort, and the preferable re-framing on live hospital discharge could not be estimated because discharge dates are available for only a minority of survivors. The re-abstraction audit indicates that this is partly a failure of capture rather than an absence in the source records: in two of the 22 records re-abstracted, the admission and discharge dates and the duration of intensive care stay were present in the source file but absent from the electronic database. Therefore, the timing of in-hospital death cannot be described, and only the overall proportion is reported. As a single-centre study, the findings may not be generalised. The study period spans changes in surgical and anaesthetic technique, disease burden, and health policy; era-stratified modelling was not undertaken because per-era samples were too small for stable estimation. Consequently, the multivariable model was not adjusted for calendar era, and the observed association between operative duration and mortality may be partially confounded by unmeasured secular changes in case mix or operative practice over time.

As a quaternary referral centre, the cohort is subject to referral and survivorship bias, because patients with hyper-acute events may not survive to reach the operating room. Standardised definitions for complications such as acute kidney injury were not consistently available in earlier years. We lacked data on time from symptom onset to intervention and on long-term survival.

### Conclusion

This retrospective observational cohort study, covering surgery performed between 1995 and 2023 at a quaternary South African centre, identifies a distinct epidemiological profile for thoracic aortic surgery, with an equal burden of dissection and aneurysm and a sub-population of young patients with traumatic rupture. In-hospital mortality was 21.9%. Prolonged operative duration was associated with mortality, although this finding should be interpreted as a marker of operative complexity rather than evidence that reducing duration alone would reduce mortality. The findings support intensive postoperative care and provide contemporary evidence on thoracic aortic surgery outcomes in sub-Saharan Africa.

## Authors’ contributions

Conceptualisation: MMM, KV, PMC

Data curation: MMM, KV

Formal analysis: MMM, MMK

Validation: AN, PMC

Methodology: MMK, PMC

Writing (original draft): MMM

Writing (review and editing): AN, KV, MMK, PMC

Supervision: PMC

## Data availability

The data are available from the corresponding author on reasonable request. Raw data cannot be made public owing to ethical restrictions and patient confidentiality.

## Funding

No funding was received for this study.

## Consent for publication

Not applicable; a waiver of individual informed consent was granted, and all data were anonymised.

## Declarations of interest

The authors declare that they have no conflicts of interest.
